# Insights into the ancestral organisation of the mammalian MHC class II region from the genome of the pteropid bat, *Pteropus alecto*

**DOI:** 10.1186/s12864-017-3760-0

**Published:** 2017-05-18

**Authors:** Justin H. J. Ng, Mary Tachedjian, Lin-Fa Wang, Michelle L. Baker

**Affiliations:** 1 CSIRO Australian Animal Health Laboratory, Health and Biosecurity Business Unit, Geelong, VIC 3220 Australia; 2 Faculty of Veterinary Science, University of Sydney, Sydney, NSW 2006 Australia; 3 Programme in Emerging Infectious Diseases, Duke-National University of Singapore Medical School, Singapore, 169857 Singapore

**Keywords:** Bat, Australian black flying-fox, *Pteropus alecto*, MHC-II, Comparative genomics

## Abstract

**Background:**

Bats are an extremely successful group of mammals and possess a variety of unique characteristics, including their ability to co-exist with a diverse range of pathogens. The major histocompatibility complex (MHC) is the most gene dense and polymorphic region of the genome and MHC class II (MHC-II) molecules play a vital role in the presentation of antigens derived from extracellular pathogens and activation of the adaptive immune response. Characterisation of the MHC-II region of bats is crucial for understanding the evolution of the MHC and of the role of pathogens in shaping the immune system.

**Results:**

Here we describe the relatively contracted MHC-II region of the Australian black flying-fox (*Pteropus alecto*), providing the first detailed insight into the MHC-II region of any species of bat. Twelve MHC-II genes, including one locus (*DRB2*) located outside the class II region, were identified on a single scaffold in the bat genome. The presence of a class II locus outside the MHC-II region is atypical and provides evidence for an ancient class II duplication block. Two non-classical loci, *DO* and *DM* and two classical, *DQ* and *DR* loci, were identified in *P. alecto*. A putative classical, *DPB* pseudogene was also identified. The bat’s antigen processing cluster, though contracted, remains highly conserved, thus supporting its importance in antigen presentation and disease resistance.

**Conclusions:**

This detailed characterisation of the bat MHC-II region helps to fill a phylogenetic gap in the evolution of the mammalian class II region and is a stepping stone towards better understanding of the immune responses in bats to viral, bacterial, fungal and parasitic infections.

**Electronic supplementary material:**

The online version of this article (doi:10.1186/s12864-017-3760-0) contains supplementary material, which is available to authorized users.

## Background

Bats are the second most species-rich group of mammals after rodents, accounting for approximately 20% of all classified living mammals [1]. Bats are in the order Chiroptera and can be further classified into two suborders; Yinpterochiroptera, which includes all of the megabats and four microbat families (Rhinopomatidae, Rhinolophidae, Hipposideridae, and Megadermatidae), and Yangochiroptera which includes all remaining microbat families [2, 3]. Relative to other species of similar body size, bats have a longer lifespan [4–8] and are one of the most successful groups of mammals having evolved to fill a variety of ecological niches across all continents, with the exception of the polar regions [1].

In recent years, bats have been increasingly recognised for their role in maintaining numerous pathogens (viruses, parasites and bacteria), with new pathogens being continually identified each year [9–18]. In addition to their association with viruses, bats are also host to a variety of other pathogens, including bacteria and parasites of zoonotic potential. A high prevalence of the bacteria, *Bartonella sp* and *Leptospira*, have been reported in a variety of bats [14, 19–21]. Intracellular hemosporidian parasites (including *Plasmodium*, *Polychromophilus*, *Nycteria*, and *Hepatocystis*) have also been identified across both suborders of bats [16]. Despite the number of pathogens that have been linked to bats, they rarely cause any clinical signs of disease in bats, a characteristic that has been hypothesised to be associated with a unique immune system [16, 22–29]. Curiously, the ability of bats to control intracellular pathogens may not extend to extracellular pathogens including some bacteria and fungi [30]. The devastating consequences of the fungal pathogen, *Pseudogymnoascus destructans* responsible for white nose syndrome (WNS), is an extreme example of an extracellular pathogen that is capable of causing disease in North American microbats [31, 32].

The MHC is the most gene-dense and polymorphic region of the genome and the majority of genes encoded within this region play a role in immune defence. The MHC region of eutherian mammals can be broadly divided into three regions, class I, class II and class III. The class I and II genes are highly polymorphic and evolve through gene duplication and conversion in response to strong selection pressure by pathogens [33, 34]. The organisation of the MHC region is highly dynamic and has been reorganised throughout vertebrate evolution as species evolve and adapt to new pathogenic and environmental pressures [35, 36]. Understanding the evolution of the MHC region has the potential to provide valuable insights into host-pathogen evolution. In most eutherian mammals studied to date, the MHC-II region is highly conserved spanning ~0.5 megabases (Mb) in pig to ~1.4 Mb in the horse genome [36–41]. The MHC-II region can be further divided into the extended and classical sub-regions, with all MHC-II genes localised in the classical sub-region [42]. Vital antigen-processing (AP) genes for the class I presentation pathway, such as proteasome subunit β types 8 and 9 (*PSMB8* and *9*), transporter associated with antigen processing 1 and 2 (*TAP1* and *2*) and Tapasin (*TAPBP*), are also found within the class II region, forming the AP gene cluster *(DOB-TAP2-PSMB8-TAP1-PSMB9-DMB-DMA-BRD2* (bromodomain - containing protein 2)*-DOA*) [38, 42]. Numerous autoimmune diseases have also been associated with genes found within the MHC-II region [43].

MHC-II molecules are heterodimers consisting of non-covalently linked α and β chains encoded by separate genes within the MHC-II region. They are expressed only on the surface of antigen presenting cells, such as B cells, monocytes, macrophages and dendritic cells, and accommodate antigens of 11 to 20 amino acid residues in length [44]. MHC-II genes can be further divided into classical and non-classical class II genes. The polymorphic classical MHC-II molecules (*DP*, *DQ* and *DR*) are responsible for the presentation of extracellular antigens (usually of bacterial, fungal and parasitic origin) to activate CD4^+^ (cluster of differentiation 4) T helper cells [45]. Activation of CD4^+^ T helper cells in turn coordinates the antibody- and cell-mediated immune responses. The non-polymorphic, non-classical class-II molecules (*DM* and *DO*) do not present antigens, but instead play an important role in antigen-processing [46].

Despite the importance of the MHC-II region in disease resistance, few studies have described MHC-II genes in bats. The limited work that has been reported to date has focused on the DRB locus of a variety of microbats and one species of megabat (*Rousettus aegyptiacus*) [47]. Extreme differences in MHC-II allelic polymorphism have been observed between different microbat species, with possible links between variation in population size, environmental and pathogen pressure [48–52]. Correlations between specific *DRB* alleles, ectoparasite load and reproductive state were also identified in the insectivorous bat, *Noctilio albiventris* [50, 53, 54].

Recently we described the class I region of the Australian black flying fox, *Pteropus alecto*, revealing a relatively condensed, yet conserved MHC-I region [55]. Comparative analysis of the bat MHC-I region provided insights into the evolution of the mammalian MHC and resulted in the identification of MHC-I genes with unique insertions in their PBG. To further build on this work and obtain deeper insights into the evolution of the MHC region, we describe the organisation of the MHC-II region of *P. alecto*, with the aid of its whole genome sequence [56]. As bats are an ancient lineage of mammals having diverged from other eutherian mammals approximately 88 million years ago (mya) [56], the bat MHC region fills an important phylogenetic gap in the evolution of the mammalian MHC. Furthermore, the identification of MHC-II genes is the first step towards understanding the role of MHC-II genes in defence against extracellular pathogens, including bacteria and parasites. To our knowledge, this is the first detailed analysis and characterisation of the MHC-II region and its content in any species of bat.

## Methods

### Bat genome data and annotation

The recently completed *P. alecto* genome was interrogated for MHC-II, AP and conserved class II flanking genes using the BLAST algorithm [57]. A single scaffold containing MHC-II related genes was re-annotated manually using GENSCAN [58] for gene prediction and gene identity confirmed using BLAST [57] against the NCBI database. The newly annotated MHC-II region was then visualised and analysed using Clone Manager Professional 9 (Sci-Ed Software, Denver CO USA) and Geneious version R7 (Biomatters Ltd, Auckland NZ).

### Comparative analysis of the bat MHC-II region and genes

The human (*Homo sapiens*), horse (*Equus caballus*) and pig (*Sus scrofa*) MHC-II regions were adapted from the Ensembl annotation (versions GRCh37.p11 for human, EquCab2 for horse and Sscrofa10.2 for pig) for comparative analysis with the bat (*P. alecto*) MHC-I region using EasyFig software [59].

### Promoter analysis

The region 600 bp upstream of human MHC-II genes (*HLA-DOA*, −*DOB*, −*DPA1*, −*DPB1*, −*DQA1*, −*DQA2*, −*DQB1*, −*DQB2*, −*DRA*, −*DRB1* and *-DRB5*) were retrieved from Ensembl (version GRCh37.p11). The corresponding region was obtained for the bat MHC-II genes. The promoter regions of the bat class II genes were analysed by comparison to the human genes. All sequences upstream from the start codon were analysed using Clone Manager Professional Version 9 software to manually identify putative promoter S-X-Y motifs. Sequences were then collated and aligned, with sequence logos [60] of the S-X-Y motifs illustrated using the Geneious version R7 software package (Available from http://www.geneious.com/).

### Gene and phylogenetic analysis

MEGA software version 5.2.1 [61] was used for all gene and phylogenetic analyses. Bat MHC-II and AP nucleotide sequences were aligned based on the protein alignment to retain codon positions with human HLA sequences as reference using MUSCLE [62]. All putative interaction sites within MHC-II genes were predicted based on Marsh et al. [63] and Bondinas et al. [64]. Corresponding nucleotide alignments were used for phylogenetic analysis using the Maximum Likelihood (ML) model with discrete Gamma distribution and 1000 bootstrap replicates [65–67]. The “Find Best Model (ML)” function was used to determine the appropriate substitution models for each dataset. The model with the lowest Bayesian Information Criterion (BIC) score was considered to best describe the substitution pattern for that dataset and was subsequently chosen for phylogenetic analysis. Neighbour Joining (NJ) [68] and Minimum Evolution (ME) [69] trees, with 1000 bootstrap replicates, were also constructed to corroborate with the ML trees. Tree Explorer was used for tree visualisation and illustration. Base-By-Base [70] was used to determine nucleotide and amino acid sequence identity between the bat and other mammalian MHC-II and AP genes.

### Accession numbers

The GenBank (http://www.ncbi.nlm.nih.gov/Genbank) accession numbers and Ensembl (http://asia.ensembl.org/index.html) transcript ID for the genes and gene products discussed in this paper are *P. alecto* scaffold202 (KB030441.1); *Homo sapiens* HLA-DMA (NM_006120); *H. sapiens* HLA-DOA (M26039); *H. sapiens* HLA-DPw3 (M27487); *H. sapiens* HLA-DQA (M26041); *H. sapiens* HLA-DRA (NM_019111); *H. sapiens* HLA-DMB (U15085); *H. sapiens* HLA-DOB (L29472); *H. sapiens* HLA-DPB (M57466); *H. sapiens* HLA-DQB (M20432); *H. sapiens* HLA-DRB4 (NM_021983); *H. sapiens* PSMB8 (ENSP00000364016); *H. sapiens* PSMB (ENST00000374859.2); *H. sapiens* TAP1 (ENST00000354258.4); *H. sapiens* TAP2 (ENST00000374897.2); *H. sapiens* TAPBP (ENST00000434618.6); *Equus caballus* DMA (HQ890199); *E. caballus* DOA (XM_005603798.2); *E. caballus* DQA (L33909); *E. caballus* DRA (JQ254083); *E. caballus* DMB (XM_005603792.2); *E. caballus* DOB (XM_005603785.2); *E. caballus* DRB (JQ254095); *E. caballus* DQB (L33910); *E. caballus* PSMB8 (ENSECAP00000016548); *E. caballus* PSMB9 (ENSECAP00000014604); *E. caballus* TAP1 (ENSECAT00000024416.1); *E. caballus* TAP2 (ENSECAP00000005757); *E. caballus* TAPBP (ENSECAP00000010701); *Sus scrofa* SLA-DMA (NM_001004039); *S. scrofa* SLA-DOA (NM_001113705); *S. scrofa* SLA-DQA1 (NM_001130224); *S. scrofa* SLA-DRA (NM_001113706); *S. scrofa* SLA-DMB (DQ431246); *S. scrofa* SLA-DQB (AY102478); *S. scrofa* SLA-DRB (AY191776); *Ovis aries* DQB1 (L08792); *Oryctolagus cuniculus* DOB (M96942); *O. cuniculus* DPB (ENSOCUT00000008716.3); *Mus musculus* DOA (M95514); *M. musculus* DQA (M21931); *M. musculus* DRA (U13648); *M. musculus* DMB (U35332); *M. musculus* PSMB8 (ENSMUSP00000025196); *M. musculus* PSMB9 (ENSMUST00000174576.2); *M. musculus* TAP1 (ENSMUST00000170086.7); *M. musculus* TAP2 (ENSMUSP00000025197); *M. musculus* TAPBP (ENSMUSP00000025161); *Rattus norvegicus* DQB (X56596); *Gallus gallus* MHC-II α chain (AY357253); *G. gallus* MHC-II β chain (M29763) and *G. gallus* DMB (AB426148).

## Results and discussion

### The bat MHC region is contracted but conserved in content

**Fig. 1:**
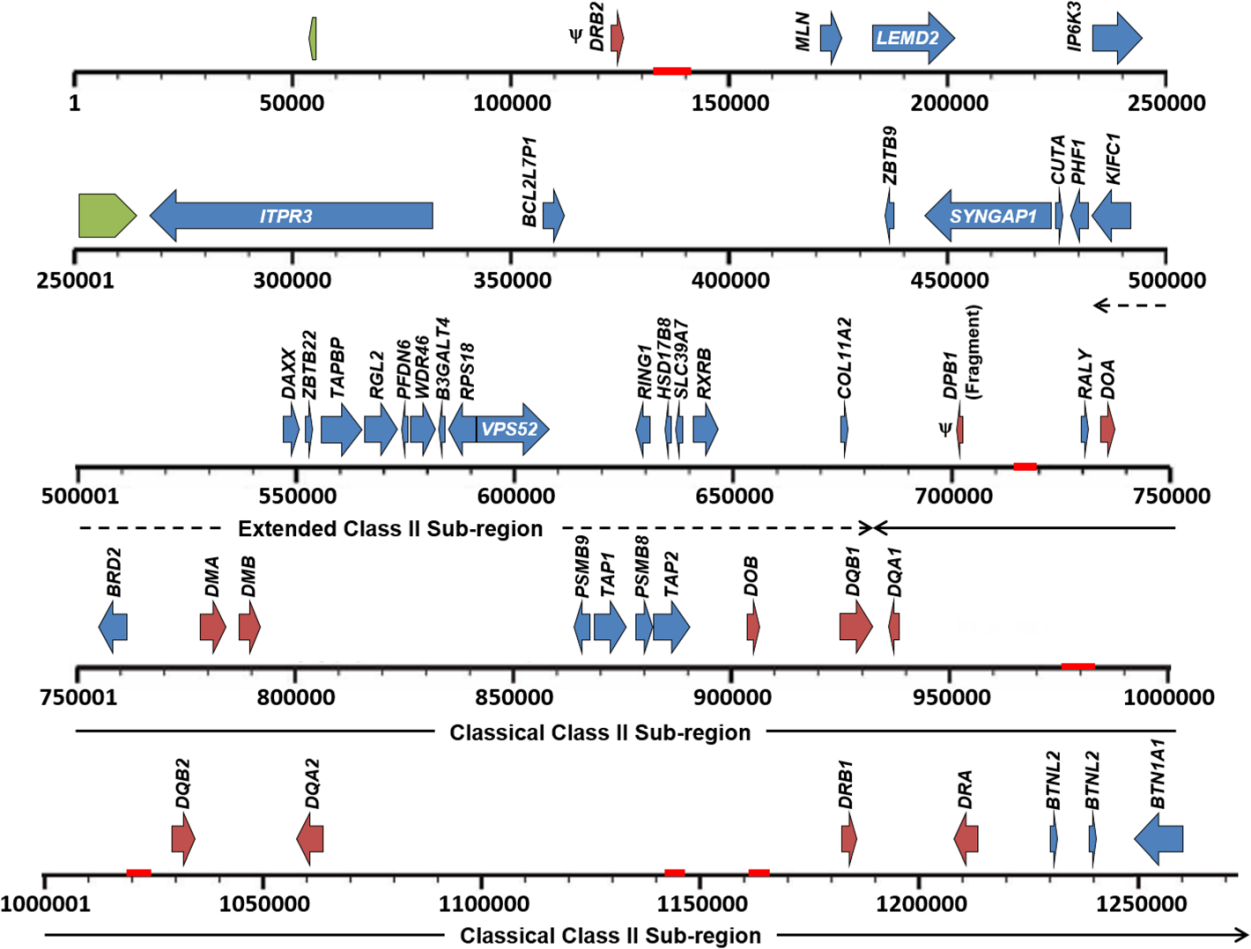
Detailed map of the bat MHC-II region (1,262,706 bp) identified on scaffold202 of the bat genome. *Maroon arrows* represent MHC-II genes, *blue arrows* represent annotated genes, and *green arrows* represent predicted, unannotated open reading frames. *Red blocks* represent gaps (>4000 bp) in the scaffold. *Dotted arrows* represent extended class II sub-region and *solid arrows* represent classical class II sub-region. ψ represents putative pseudogenes

The mammalian MHC-II region is divided into a classical region containing all of the MHC-II genes and an extended sub-region containing antigen processing genes [42]. A single scaffold (scaffold202) spanning 5,012,706 basepairs (bp) identified in the *P. alecto* genome [56] contained a 1,262,706 bp region corresponding to the entire MHC-II region. A total of 47 loci including 12 MHC-II genes, five AP genes and 30 other genes were identified, spanning from ~360 kilobases (kb) upstream of the extended class II subregion to the end of the classical class II subregion (Fig. 1). Two open reading frames (ORFs) with no homologues were also predicted. The coordinates and accession numbers of the predicted genes are summarised in Table 1. Genes were annotated based on their similarity to orthologous genes in other species. MHC-II genes were named according to the nomenclature proposed by Klein et al. [71] and their evolutionary relationships with MHC-II families (described in the phylogenetic analysis below)*.*

**Table 1:** List of annotated genes in Bat MHC-II region

Description	Gene	Start	End	Strand	Accession	Locus Tag^a^
NA	-	54622	54407	-	ELK16616	PAL_GLEAN10007084
ψ MHC Class II DR β chain 2	*DRB2*	124020	127163	+	ELK16617	PAL_GLEAN10007085
Promotilin	*MLN*	171900	176944	+	ELK16618	PAL_GLEAN10007086
LEM domain-containing protein 2	*LEMD2*	184083	200005	+	ELK16619	PAL_GLEAN10007087
Inositol hexakisphosphate kinase 3	*IP6K3*	233132	244985	+	ELK16620	PAL_GLEAN10007088
Uncharacterised protein C6orf125 homolog	-	251970	264591	+	ELK16621	PAL_GLEAN10007089
Inositol 1,4,5-trisphosphate receptor type 3	*ITPR3*	330989	267163	-	ELK16622	PAL_GLEAN10007090
Putative Bcl-2 homologous antagonist/killer 2	*BCL2L7P1*	358514	361666	+	ELK16623	PAL_GLEAN10007091
Zinc finger and BTB domain-containing protein 9	*ZBTB9*	435884	437284	-	ELK16624	PAL_GLEAN10007093
Ras GTPase-activating protein SynGAP	*SYNGAP1*	444762	473798	-	ELK16625	PAL_GLEAN10007094
Protein CutA	*CUTA*	475923	477426	+	ELK16626	PAL_GLEAN10007095
PHD finger protein 1	*PHF1*	483072	477988	-	ELK16627	PAL_GLEAN10007096
Kinesin-like protein KIFC1	*KIFC1*	493984	483931	-	ELK16628	PAL_GLEAN10007097
Death domain-associated protein 6	*DAXX*	547565	550867	+	ELK16629	PAL_GLEAN10007098
Zinc finger and BTB domain-containing protein 22	*ZBTB22*	552438	554336	+	ELK16630	PAL_GLEAN10007099
Tapasin	*TAPBP*	555260	564216	+	ELK16631	PAL_GLEAN10007100
Ral guanine nucleotide dissociation stimulator-like 2	*RGL2*	566433	572764	+	ELK16632	PAL_GLEAN10007101
Prefoldin subunit 6	*PFDN6*	575085	574122	-	ELK16633	PAL_GLEAN10007102
WD repeat-containing protein 46	*WDR46*	575753	582904	+	ELK16634	PAL_GLEAN10007103
Beta-1,3-galactosyltransferase 4	*B3GALT4*	585083	583725	-	ELK16635	PAL_GLEAN10007104
40S ribosomal protein S18	*RPS18*	590495	585718	-	ELK16636	PAL_GLEAN10007105
Vacuolar protein sorting-associated protein 52 homolog	*VPS52*	590542	608895	+	ELK16637	PAL_GLEAN10007106
E3 ubiquitin-protein ligase RING1	*RING1*	632961	629730	-	ELK16638	PAL_GLEAN10007107
Estradiol 17-beta-dehydrogenase 8	*HSD17B8*	636805	635286	-	ELK16639	PAL_GLEAN10007108
Zinc transporter SLC39A7	*SLC39A7*	640599	638121	-	ELK16640	PAL_GLEAN10007109
Retinoic acid receptor RXR-beta	*RXRB*	641371	646657	+	ELK16641	PAL_GLEAN10007110
Collagen alpha-2(XI) chain	*COL11A2*	674830	675093	+	ELK16642	PAL_GLEAN10007112
ψMHC Class II DP β chain 1 (Fragment)	*DPB1*	703197	701801	-	ELK16643	PAL_GLEAN10007114
RNA-binding protein Raly	*RALY*	730049	730573	+	ELK16644	PAL_GLEAN10007116
MHC Class II DO α chain	*DOA*	734481	736943	+	ELK16645	PAL_GLEAN10007117
Bromodomain-containing protein 2	*BRD2*	762249	755609	-	ELK16646	PAL_GLEAN10007118
MHC Class II DM α chain	*DMA*	779647	782542	+	ELK16647	PAL_GLEAN10007119
MHC Class II DM β chain	*DMB*	788588	791667	+	-	-
Proteasome subunit beta type-9	*PSMB9*	868896	864164	-	ELK16648	PAL_GLEAN10007120
Antigen peptide transporter 1	*TAP1*	869430	876791	+	ELK16649	PAL_GLEAN10007121
Proteasome subunit beta type-8	*PSMB8*	878304	881075	+	ELK16650	PAL_GLEAN10007122
Antigen peptide transporter 2	*TAP2*	883417	891866	+	ELK16651	PAL_GLEAN10007123
MHC Class II DO β chain	*DOB*	904258	907879	+	ELK16652	PAL_GLEAN10007124
MHC Class II DQ β chain 1	*DQB1*	924949	931213	+	ELK16653	PAL_GLEAN10007125
MHC Class II DQ α chain 1	*DQA1*	948995	947759	-	-	PAL_GLEAN10007126
MHC Class II DQ β chain 2	*DQB2*	1039500	1044135	+	-	-
MHC Class II DQ α chain 2	*DQA2*	1064301	1059023	-	ELK16654	PAL_GLEAN10007127
MHC Class II DR β chain 1	*DRB1*	1184173	1187159	+	-	PAL_GLEAN10007128
MHC Class II DR α chain	*DRA*	1213079	1209726	-	ELK16655	PAL_GLEAN10007129
Butyrophilin-like protein 2	*BTNL2*	1230144	1232101	+	ELK16656	PAL_GLEAN10007130
Butyrophilin-like protein 2	*BTNL2*	1239555	1241047	+	ELK16657	PAL_GLEAN10007131
Butyrophilin subfamily 2 member A3	*BTN1A1*	1260527	1250684	-	ELK16658	PAL_GLEAN10007132

^a^Locus tags refer to annotations in the *P. alecto* whole genome

ψ represents putative pseudogenes

The bat classical and extended MHC-II sub-regions, bordered by butyrophilin-like protein 2 (*BTNL2*) and kinesin family member C1 (*KIFC1*), were highly contracted, spanning ~0.78 Mb compared to ~1.3 – 2.2 Mb in other mammals [38, 39, 42, 72, 73]. The pig (*Sus scrofa*) is the only other mammalian species known to have a contracted MHC-II region (~0.5 Mb) [41, 74]. A similar pattern has previously been described for the MHC I region, which is contracted in both bats and pigs [55]. The contracted MHC region of bats and pigs is consistent with their smaller genome sizes; ~2.3 gigabases (Gb) for bats and ~2.7 Gb for pigs compared to humans and other mammals, which have an average genome size of ~3.5 Gb [56, 75].

**Fig. 2:**
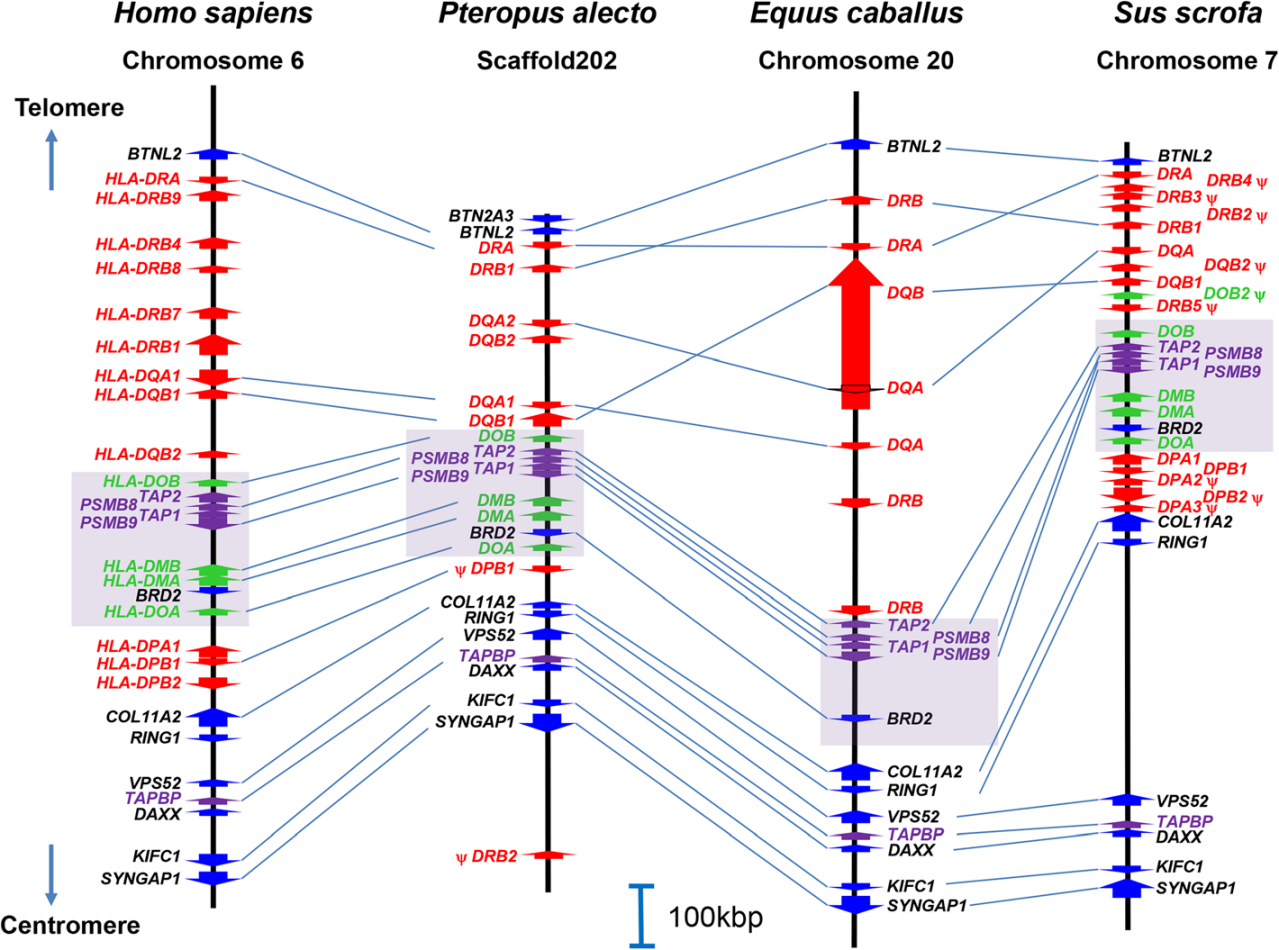
Comparative gene maps of bat MHC-II region (centre) against human, horse and pig MHC-II regions. *Red arrows* represent classical MHC-II genes, *green arrows* represent non-classical MHC-II genes, *blue arrows* represent flanking MHC-II region genes and *purple arrows* represent essential AP genes [37]. The areas highlighted in *purple* represent the antigen-processing (AP) cluster. ψ represents putative pseudogenes. The direction and orientation of the MHC-II regions relative to the telomere centromere are shown. The human, horse and pig gene maps were adapted from the Ensembl annotation

The organisation of the bat MHC-II region was compared with corresponding regions from the human, horse and pig genomes. The human MHC region was used as a reference since it is the most well characterised, the horse was included due to its close phylogenetic relationship with bats, and pig was included due the similarity in size of its MHC to that of bats [56, 76]. The organisation of the bat MHC-II region is highly syntenic with the human, horse and pig MHC-II regions (Fig. 2). The mammalian classical class II sub-region, between reference framework genes *BTNL2* and collagen, type XI, alpha 2 (*COL11A2*), is highly conserved in eutherians and contains all of the MHC class II genes [36, 37, 40]. Although this region is highly contracted in *P. alecto*, the gene order and content remain conserved, including the presence of the entire AP gene cluster *(DOB-TAP2-PSMB8-TAP1-PSMB9-DMB-DMA-BRD2-DOA*). Eleven MHC-II genes were found within the bat classical class II sub-region, compared to 16 in human [38, 42], seven in horse [39] and 19 in pig [41]. The extended sub-region gene organisation, between reference framework genes *COL11A2* and *KIFC1*, is also highly conserved, with contraction observed in the bat (~200 kb) compared with human (~250 kb), horse (~250 kb) and pig (~400 kb). The essential AP gene, *TAPBP*, was also highly conserved in terms of location and orientation.

**Fig. 3:**
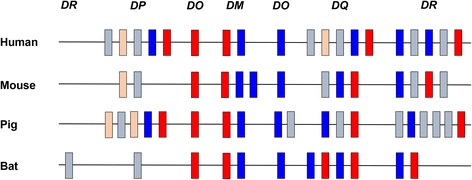
Simplified genomic maps of MHC-II genes in human, mouse, pig and bat. *Red* and *blue boxes* represent α and β chains respectively. *Light red* and *light blue boxes* represent pseudogenes for α and β chains respectively. Only human, mouse and pig were included as the MHC-II region of these species has been well characterised (The MHC Sequencing Consortium 1999; Velten et al. 1999; Kumánovics et al. 2003; Horton et al. 2004; Renard et al. 2006)

The bat class II region contains all of the known classical class II gene families; *DP*, *DQ* and *DR* that are responsible for antigen presentation. The *DM* and *DO* genes, that encode non-classical class II molecules and play a vital role in peptide loading onto classical class II molecules, were also present in the bat MHC-II region. Bats appear to lack functional *DP* α and β chains, with only a single *DPB1* locus encoding a partial *DP* β chain sequence, likely corresponding to a pseudogene (Fig. 3). The significance of this finding has yet to be elucidated. However, the overall genetic diversity of the *DP* loci in mammals are generally lower compared to their *DQ* and *DR* counterparts (human, IPD – IMGT/HLA v3.27; pig [74]). The bat *DP* locus may historically have been under less selective pressure, contributing to lower *DP* diversity [34]. Alternatively, low recombination within the bat MHC-II region may have resulted in fewer *DP* loci compared to other mammals [33]. As illustrated in Fig. 3, pairs of MHC-II genes are encoded adjacent to each other, with the exception of *DOA* and *DOB*. Horse was omitted from this analysis due to incomplete annotation of its MHC-II genes. The location of essential non-classical class II genes, *DM* and *DO*, are also well conserved in the bat. An intriguing finding is that *P. alecto* possesses two potentially functional copies of *DQA* and *DQB*. This finding is consistent with previous observations of multiple *DQ* genes in herbivores, including horses [39], sheep [73] and pandas [77], but not in non-herbivorous mammals, such as humans, pigs and dogs. Wan et al. [77] speculated a possible link between multiple functional *DQ* loci and herbivory. Possession of a larger repertoire of functional *DQ* loci in herbivores could potentially confer disease resistance to bacterial, fungal and parasitic infection, which are encountered more frequently through ingestion of plants than meat [77].

An unusual finding was the identification of an MHC-II gene outside the MHC-II region. A gene designated *DRB2* was located ~355 kb upstream of SYNGAP1 which marks the end of the extended class II sub-region in other species and ~580 kb from the nearest class II gene (*DPB*) (Fig. 1). Comparative analysis of the composition of class II region of *P. alecto* with that of other mammals illustrates the unusual nature of the presence of a class II gene (light blue block) in this location in the bat genome (Fig. 3). An MHC-II gene located outside the conserved framework genes *COL11A2* and *BTNL2* has not been described in any other eutherian mammal to date. However, differences in the organisation of the class II region have been reported in marsupials. The opossum has a combined class I/II region and tammar wallaby class II genes are located in two regions of the genome, thus providing evidence for the presence of class II genes outside the class II region prior to the divergence of marsupials and eutherians [72, 78]. The bat *DRB2* gene appears to be a pseudogene and may therefore be a remnant of an ancient class II duplication block that became extinct during mammalian evolution.

### Bat MHC-II genes

Ten bat class II genes (*Ptal-DMA*, −*DOA*, −*DQA1*, −*DQA2*, −*DRA*, −*DMB*, −*DOB*, −*DQB1*, −*DQB2* and *-DRB1*) appear to be functional based on the presence of intact ORFs. Two of the loci have been classified as pseudogenes, due to an incomplete ORF in the case of *DPB1* or the presence of stop codons within the ORF for *DRB2*, which is within the range of two pseudogenes in pigs to eight in humans. Five MHC-IIA and four MHC-IIB transcripts previously identified in a *P. alecto* transcriptome dataset [76] corresponded to MHC-II genes identified in this study, thus providing further evidence that these loci encode functional genes (Additional file 1).

Alignments of deduced protein sequences of bat MHC-IIA and IIB genes, with sequences from other mammals, revealed the presence of conserved cysteine residues, peptide-binding and CD4 interaction sites in the bat class II proteins (Additional file 2 and 3 respectively). The conserved location of cysteine residues responsible for intra-chain disulphide bonds in the bat class II sequences is consistent with conservation of the 3D structure with human class II molecules [44].

### Sequence similarity and phylogenetic analysis of bat MHC-II genes

Overall, the bat MHC-IIA and -IIB genes are highly conserved with those of other mammals (Additional file 2B). The α1 and β1 domains which form the antigen binding region shared consistently lower nucleotide similarity compared to the α2 and β2 domains (76 – 98 and 61 – 92% respectively), reflecting the evolution of the antigen binding region in response to pathogen pressure (Additional files 2 and 3). *DOB* was the only exception, sharing higher sequence similarity in the β1 domain compared to the β2 domain (Additional file 3B). In humans and mice, DO negatively regulates DM by stably associating with DM to inhibit peptide loading, thus affecting the peptide repertoire presented to T cells [79]. The high conservation of the bat *DOB* β1 domain may reflect a similar role for this molecule in *P. alecto*. Amino acid similarity followed similar trends to those described above for nucleotide similarity across the α2 and β2 domains.

**Fig. 4:**
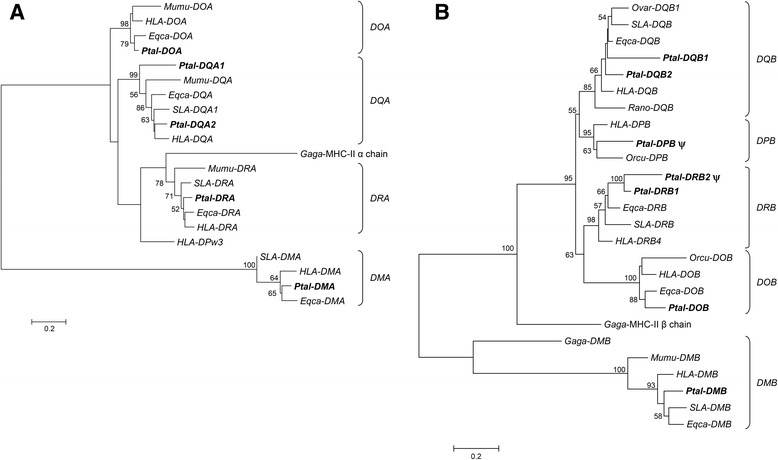
Phylogenetic trees of MHC-II genes. **a** MHC-II A gene Maximum likelihood phylogeny based on alignment of nucleotide sequences corresponding to the α1 and α2 domains. **b** MHC-II B gene Maximum likelihood phylogeny based on nucleotide sequences corresponding to the β1 and β2 domains. A discrete Gamma distribution was used to model evolutionary rate differences among sites (5 categories (+*G*, parameter = 1.5470)). The tree is drawn to scale, with branch lengths representing the number of substitutions per site. Branch support is indicated as percentage of trees out of 1000 bootstrap replicates that produce the same branching order. ψ represents putative pseudogenes. *HLA* – human leucocyte antigen; *SLA* – swine leucocyte antigen; *Eqca* – *Equus caballus*; *Ovar* – *Ovis aries*; *Mumu* – *Mus musculus*; *Orcu* – *Oryctolagus cuniculus*; *Gaga* – *Gallus gallus*

MHC-II genes evolve via the birth-and-death process [34] but their gene turnover is much slower compared to their class I counterparts, resulting in orthologous relationships between class II genes from different species [33]. Phylogenetic analysis of the five bat class IIA genes (α subunit; Fig. 4a) and seven bat class IIB genes (β subunit; Fig. 4b) were performed using exon 3 (α2 or β2 domains) nucleotide sequences to include all functional genes and putative pseudogenes. Since the α2 and β2 domains are not involved in antigen presentation, they evolve in the absence of pathogen selection pressures, thus providing the most accurate representation of the phylogenetic relationships of MHC-II genes. As shown in Fig. 4, the bat class II genes formed orthologous relationships with corresponding class II genes from other species. The bat *DMA*, *DOA*, *DOB*, *DRB1* and *DRB2* genes were most closely related to the horse as expected, the two sharing a common ancestor ~88 mya [56]. The remaining bat class II genes cluster with corresponding sequences from the laurasiatherian mammals (Fig. 4). This result concurred with phylogenetic trees in Kupfermann et al. (1999), demonstrating MHC-II intron sequences from two species of microbats and one megabat cluster in a species specific manner, similar to other mammals. Interestingly, *Ptal-DQA1* is basal to *Ptal-DQA2* and other mammalian *DQA* genes analysed, possibly reflecting its ancestral nature and the evolution of *Ptal-DQA2* through a recent duplication event.

### Analysis of the bat MHC-II promoter region

The transcription of MHC-II genes is regulated by conserved sequences in the proximal promoter regions (S, X and Y boxes) [80, 81]. These motifs are highly conserved among class II loci, both within and across mammalian species, and are necessary for optimal constitutive and cytokine induced gene expression. Transcription is largely regulated by the class II transactivator (*CIITA*), which interacts with several transcription factors, particularly those that bind to the SXY motif [82]. To identify regulatory motifs in the bat class II sequences, the region 500 bp upstream of the translation start site of the 12 bat MHC-II genes was analysed. Using manual examination and annotation, with reference to other mammalian promoter elements, the S-X-Y motifs were identified in seven of the 12 bat class II genes (*Ptal-DMA*, −*DOA*, −*DOB*, −*DQA2*, −*DQB1*, −*DQB2* and *-DRA*), with coordinates within the class II genomic region summarised in Table 2. No S-X-Y motifs could be identified for *Ptal-DMB*, −*DPB1*, −*DQA1*, −*DRB1* or *-DRB2*. No CAAT or TATA boxes were identified in any of the bat MHC-II genes, similar to class II genes from the Tasmanian devil [83]. Although putative CAAT and TATA boxes are present in human MHC-II genes, their functional significance is unknown [81]. Successful identification of putative promoter elements provides further evidence that at least seven of the bat class II genes are likely functional and is consistent with evidence for the presence of two pseudogenes, *DRB2* and *DPB1*.

**Table 2:** Coordinates of Bat class II S-X-Y motifs within the MHC-II genomic region on scaffold202

Gene Name	Strand	Gene Start	S-X-Y Start	S-X-Y End	S-X-Y Relative Position (S-X-Y End to Gene Start)
*DMA*	+	779647	779475	779555	−92
*DMB*	+	788588	Not found	Not found	n/a
*DOA*	+	734481	734305	734363	−118
*DOB*	+	904258	904074	904121	−137
*DPB1 *ψ	-	703197	Not found	Not found	n/a
*DQA1*	-	Not found	Not found	Not found	n/a
*DQA2*	-	1064301	1064483	1064419	118
*DQB1*	+	924949	924738	924799	−150
*DQB2*	+	1039500	1039285	1039348	−152
*DRA*	-	1213079	1213260	1213211	132
*DRB1*	+	Not found	Not found	Not found	n/a
*DRB2 *ψ	+	Not found	Not found	Not found	n/a

ψ represents putative pseudogenes

**Fig. 5:**
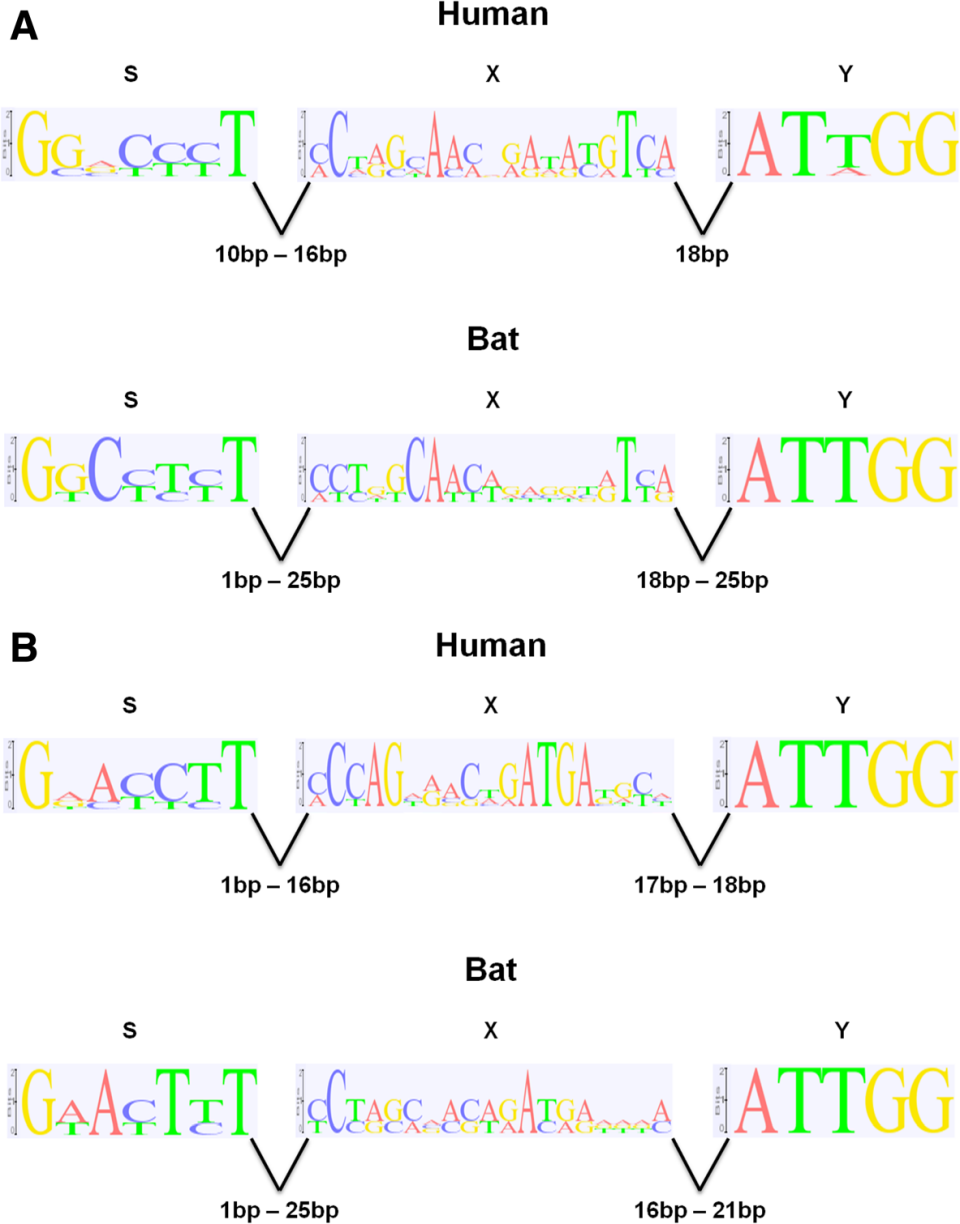
Comparison of the S-X-Y motifs in the (**a**) MHC-II A genes and (**b**) MHC-II B genes of bat and human. Logos of corresponding position-specific scoring matrix models are presented. The height, in terms of log_2_, of each stack of symbols (*y-axis*) represents information content in each position of the DNA sequence (*x-axis*), with a maximum value of 2

Using the 11 human MHC-II genes (*HLA-DOA*, −*DOB*, −*DPA1*, −*DPB1*, −*DQA1*, −*DQA2*, −*DQB1*, −*DQB2*, −*DRA*, −*DRB1* and *-DRB5*) for comparison, sequence logo diagrams of the S-X-Y motifs of the bat and human class II genes were generated (Fig. 5). The distance between the S and X motifs ranged from 1 to 25 bp in the bat α and β chains compared to 1 to 16 bp in the human class II genes. In human B cells, class II genes with greater distance between S and X motifs have higher constitutive expression [84]. Whether the greater S-X distance observed in the bat genes is indicative of higher expression in bat cells remains to be determined. The X and Y motifs interact with DNA-binding heterotrimers RFX [85] and NF-Y [86, 87] respectively, which are subsequently bound by CIITA acting as a master regulator of MHC-II expression [88]. The distance between the X and Y motifs in the bat, which ranged from 16 to 25 bp, falls within that of human class II genes of 17 to 18 bp, consistent with bats potentially using similar factors (transcriptional enhancers or repressors) to regulate their class II gene expression.

### Analysis of the Bat Antigen-Processing (AP) genes

The AP gene cluster *(DOB-TAP2-PSMB8-TAP1-PSMB9-DMB-DMA-BRD2-DOA*) resides within the bat classical class II sub-region as discussed above (Figs. 1 and 2). Although this sub-region is considered to be highly polymorphic due to its MHC-II gene content, the organisation of the AP cluster is highly conserved across most mammals [36, 37, 40, 42, 72, 77, 89]. The bat AP gene cluster is contracted compared to that of humans (~180 kb vs ~200 kb respectively), but remains highly conserved in synteny (Fig. 2). This conservation is essential in ensuring successful cleavage of peptides by PSMBs and subsequent peptide transportation for processing by TAPs via the class I presentation pathway. Detailed analysis of bat AP genes (*PSMB8*, *PSMB9*, *TAP1*, *TAP2* and *TAPBP*) within the class II region revealed high genetic conservation compared with other mammals. Sequence alignments of AP genes indicated high nucleotide and amino acid similarity (Additional file 4) with sequences from other mammalian species. This would suggest that AP genes in bats are functional, therefore ensuring proper processing and loading of peptides onto bat MHC-I molecules. Together, these results are consistent with the presence of both functionally and structurally conserved bat MHC-II molecules, with similar antigen-presenting capabilities and properties to those found in other mammals.

## Conclusion

The bat MHC-II region is condensed in size but highly conserved with that of other eutherian mammals. At least 12 MHC-II genes are present in the bat genome, two of which (*DPB1* and *DRB2*) appear to be pseudogenes, with the *DPA* locus remaining elusive. All identified bat class II loci have relatively conserved gene structures and were orthologous to other mammalian class II loci. Bats also possess an atypical MHC-II region, with at least one MHC-II gene (*DRB2*) located outside the class II region. The presence of a class II gene outside the MHC-II region is a first for a eutherian mammal and provides evidence for an ancient class II duplication block. This first resolution of the bat MHC-II region contributes valuable information on the comparative genomic evolution of the mammalian immune system. Detailed analysis of AP genes further suggests highly conserved and functional MHC-I and -II antigen presentation pathways, supporting the importance of this region in disease resistance in general. The characterisation of genes within the MHC-II region provides the first step towards understanding their roles in resistance to disease in response to the high diversity of viruses, bacteria and parasites identified in bats. Further studies to examine the presentation of peptides associated with extracellular pathogens by MHC-II molecules may contribute to our understanding of the response of bats to infection with extracellular pathogens. This study also provides the basis for further characterisation of the diversity of individual MHC-II genes in *P. alecto* and the role of selective pressures, including pathogens, in shaping their diversity. Detailed characterisation of MHC-II regions from different bat species will also be required to determine whether the architectural pattern observed in *P. alecto* applies across other bat species.

## Additional files

Additional file 1: Previously Reported *P. alecto* MHC-II Transcriptome Sequences and Their Correspondence to Loci. (DOC 33 kb)

Additional file 2: Amino acid alignment of bat (*Ptal*) MHC-II A genes, (A) *DMA*, (B) *DOA*, (C) *DQA* and (D) *DRA*, against various mammals. Dashes indicate identical residues; Dots indicate gaps; P Peptide-Binding Sites; 4 CD4 Interaction Sites; C Cysteine residues in α2 domain likely to form intra-chain disulphide bonds. Percentage similarity of sequences within a particular α domain is reflected at the end of their respective sequences, with reference to the top sequence in the alignment. Red and blue represent percentage similarity of nucleotides and amino acid residues respectively within the stated α1 and α2 domains. *Hosa – Homo sapiens*; *Eqca* – *Equus caballus; Susc – Sus scrofa; Mumu* – *Mus musculus*. (DOC 412 kb)

Additional file 3: Amino acid alignment of bat (*Ptal*) MHC-II B genes, (A) *DMB*, (B) *DOB*, (C) *DPB*, (D) *DQB* and (E) *DRB*, against various vertebrates. Dashes indicate identical residues; Dots indicate gaps; P Peptide-Binding Sites; 4 CD4 Interaction Sites; C Cysteine residues in β1 and β2 domains likely to form intra-chain disulphide bonds. Percentage similarity of sequences within a particular β domain is reflected at the end of their respective sequences, with reference to the top sequence in the alignment. Red and blue represent percentage similarity of nucleotides and amino acid residues respectively within the stated β1 and β2 domains. *Hosa – Homo sapiens*; *Eqca* – *Equus caballus; Susc – Sus scrofa; Mumu* – *Mus musculus; Ovar* – *Ovis aries*; *Orcu* – *Oryctolagus cuniculus*; *Gaga* – *Gallus gallus*. (DOC 364 kb)

Additional file 4: Amino acid alignment of bat (*Ptal*) antigen-processing (AP) genes, (A) *PSMB8*, (B) *PSMB9*, (C) *TAP1*, (D) *TAP2* and (E) *TAPBP*, against human (*Hosa*), mouse (*Mumu*) and horse (*Eqca*). Dashes indicate identical residues; Dots indicate gaps. Percentage similarity of sequences is reflected at the end of their respective sequences, with reference to the top sequence (*Ptal*) in the alignment. Red and blue represent percentage similarity of nucleotides and amino acid residues respectively. (DOC 434 kb)

## Abbreviations

AP: Antigen-processing
BIC: Bayesian Information Criterion
bp: Base pairs
*BRD2*: Bromodomain-containing protein 2
*BTNL2*: Butyrophilin-like protein 2
CD4: Cluster of differentiation 4
*CIITA*: Class II transactivator
*COL11A2*: Collagen, type XI, alpha 2
DNA: Deoxyribonucleic acid
Gb: Gigabases
*HLA*: Human leukocyte antigen
kb: Kilobases
*KIFC1*: Kinesin family member C1
Mb: Megabases
ME: Minimum evolution
MHC: Major histocompatibility complex
MHC-II: Class II MHC
ML: Maximum Likelihood
mya: Million years ago
NJ: Neighbour Joining
ORFs: Open reading frames
*PSMB*: Proteasome subunit β
*TAP*: Transporter associated with antigen processing
*TAPBP*: Tapasin

## References

[1] Corbet GB, Hill JE (1980). A World List of Mammalian Species.

[2] Simmons NB, Wilson DE, Reeder DM (2005). Order chiroptera. Mammal Species of the World: A Taxonomic and Geographic Reference, 3rd Edition.

[3] Teeling EC, Springer MS, Madsen O, Bates P, O’Brien SJ, Murphy WJ (2005). A Molecular Phylogeny for Bats Illuminates Biogeography and the Fossil Record. Science.

[4] Bourliere F (1958). The Comparative Biology of Aging. J Gerontol.

[5] Herreid CF (1964). Bat longevity and metabolic rate. Exp Gerontol.

[6] Wilkinson GS, South JM (2002). Life history, ecology and longevity in bats. Aging Cell.

[7] Podlutsky AJ, Khritankov AM, Ovodov ND, Austad SN (2005). A New Field Record for Bat Longevity. J Gerontol Ser A Biol Med Sci.

[8] Munshi-South J, Wilkinson GS (2010). Bats and birds: Exceptional longevity despite high metabolic rates. Ageing Res Rev.

[9] Halpin K, Young PL, Field H, Mackenzie JS (1999). Newly discovered viruses of flying foxes. Vet Microbiol.

[10] Jia GL, Zhang Y, Wu TH, Zhang SY, Wang YN (2003). Fruit bats as a natural reservoir of zoonotic viruses. Chin Sci Bull.

[11] Calisher CH, Childs JE, Field HE, Holmes KV, Schountz T (2006). Bats: Important reservoir hosts of emerging viruses. Clin Microbiol Rev.

[12] Wong S, Lau S, Woo P, Yuen KY (2007). Bats as a continuing source of emerging infections in humans. Rev Med Virol.

[13] Drexler JF, Corman VM, Muller MA, Maganga GD, Vallo P, Binger T, Gloza-Rausch F, Rasche A, Yordanov S, Seebens A (2012). Bats host major mammalian paramyxoviruses. Nat Commun.

[14] Mühldorfer K (2013). Bats and Bacterial Pathogens: A Review. Zoonoses Public Health.

[15] Jones KE, Patel NG, Levy MA, Storeygard A, Balk D, Gittleman JL, Daszak P (2008). Global trends in emerging infectious diseases. Nature.

[16] Schaer J, Perkins SL, Decher J, Leendertz FH, Fahr J, Weber N, Matuschewski K (2013). High diversity of West African bat malaria parasites and a tight link with rodent Plasmodium taxa. Proc Natl Acad Sci.

[17] Wu Y, Wu Y, Tefsen B, Shi Y, Gao GF (2014). Bat-derived influenza-like viruses H17N10 and H18N11. Trends Microbiol.

[18] Brook CE, Bai Y, Dobson AP, Osikowicz LM, Ranaivoson HC, Zhu Q, Kosoy MY, Dittmar K (2015). Bartonella spp. in Fruit Bats and Blood-Feeding Ectoparasites in Madagascar. PLoS Negl Trop Dis.

[19] Veikkolainen V, Vesterinen EJ, Lilley TM, Pulliainen AT (2014). Bats as Reservoir Hosts of Human Bacterial Pathogen, Bartonella mayotimonensis. Emerg Infect Dis.

[20] Lei BR, Olival KJ (2014). Contrasting Patterns in Mammal–Bacteria Coevolution: *Bartonella* and *Leptospira* in Bats and Rodents. PLoS Negl Trop Dis.

[21] Kosoy M, Bai Y, Lynch T, Kuzmin IV, Niezgoda M, Franka R, Agwanda B, Breiman RF, Rupprecht CE (2010). Bartonella spp. in Bats, Kenya. Emerg Infect Dis.

[22] Sulkin SE, Allen R, Sims R, Singh KV (1966). Studies of Arthropod-Borne Virus Infections in Chiroptera: IV. The Immune Response of the Big Brown Bat (Eptesicus f. fuscus) Maintained at Various Environmental Temperatures to Experimental Japanese B Encephalitis Virus Infection. AmJTrop Med Hyg.

[23] Swanepoel R, Leman PA, Burt FJ, Zachariades NA, Braack LEO, Ksiazek TG, Rollin PE, Zaki SR, Peters CJ (1996). Experimental Inoculation of Plants and Animals with Ebola Virus. Emerg Infect Dis.

[24] Williamson MM, Hooper PT, Selleck PW, Gleeson LJ, Daniels PW, Westbury HA, Murray PK (1998). Transmission studies of Hendra virus (equine morbilli-virus) in fruit bats, horses and cats. Aust Vet J.

[25] Williamson MM, Hooper PT, Selleck PW, Westbury HA, Slocombe RF (2000). Experimental Hendra Virus Infection in Pregnant Guinea-pigs and Fruit Bats (*Pteropus poliocephalus*). J Comp Pathol.

[26] Leroy EM, Kumulungui B, Pourrut X, Rouquet P, Hassanin A, Yaba P, Delicat A, Paweska JT, Gonzalez JP, Swanepoel R (2005). Fruit bats as reservoirs of Ebola virus. Nature.

[27] Leroy EM, Epelboin A, Mondonge V, Pourrut X, Gonzalez J-P, Muyembe-Tamfum J-J, Formenty P (2009). Human Ebola Outbreak Resulting from Direct Exposure to Fruit Bats in Luebo, Democratic Republic of Congo, 2007. Vector-Borne Zoonotic Dis.

[28] Middleton DJ, Morrissy CJ, van der Heide BM, Russell GM, Braun MA, Westbury HA, Halpin K, Daniels PW (2007). Experimental Nipah Virus Infection in Pteropid Bats (Pteropus poliocephalus). J Comp Pathol.

[29] Halpin K, Hyatt AD, Fogarty R, Middleton D, Bingham J, Epstein JH, Rahman SA, Hughes T, Smith C, Field HE, Daszak P (2011). Pteropid Bats are Confirmed as the Reservoir Hosts of Henipaviruses: A Comprehensive Experimental Study of Virus Transmission. Am J Trop Med Hyg.

[30] Brook CE, Dobson AP: Bats as ‘special’ reservoirs for emerging zoonotic pathogens. Trends Microbiol. 2015;23(3):172-180. 10.1016/j.tim.2014.12.004 PMC7126622 25572882

[31] Gargas A, Trest MT, Christensen M, Volk TJ, Blehert DS (2009). Geomyces destructans sp. nov. associated with Bat white-nose syndrome. Mycotaxon.

[32] Lorch JM, Meteyer CU, Behr MJ, Boyles JG, Cryan PM, Hicks AC, Ballmann AE, Coleman JTH, Redell DN, Reeder DM, Blehert DS (2011). Experimental infection of bats with Geomyces destructans causes white-nose syndrome. Nature.

[33] Hughes AL, Nei M (1990). Evolutionary relationships of class II major-histocompatibility-complex genes in mammals. Mol Biol Evol.

[34] Nei M, Gu X, Sitnikova T (1997). Evolution by the birth-and-death process in multigene families of the vertebrate immune system. Proc Natl Acad Sci.

[35] Kulski JK, Shiina T, Anzai T, Kohara S, Inoko H (2002). Comparative genomic analysis of the MHC: the evolution of class I duplication blocks, diversity and complexity from shark to man. Immunol Rev.

[36] Kelley J, Walter L, Trowsdale J (2005). Comparative genomics of major histocompatibility complexes. Immunogenetics.

[37] Trowsdale J (1995). “Both man & bird & beast”: comparative organization of MHC genes. Immunogenetics.

[38] The MHC sequencing consortium (1999). Complete sequence and gene map of a human major histocompatibility complex. Nature.

[39] Gustafson AL, Tallmadge RL, Ramlachan N, Miller D, Bird H, Antczak DF, Raudsepp T, Chowdhary BP, Skow LC (2003). An ordered BAC contig map of the equine major histocompatibility complex. Cytogenet Genome Res.

[40] Kumánovics A, Takada T, Lindahl KF (2003). Genomic organization of the mammalian MHC. Annu Rev Immunol.

[41] Renard C, Hart E, Sehra H, Beasley H, Coggill P, Howe K, Harrow J, Gilbert J, Sims S, Rogers J (2006). The genomic sequence and analysis of the swine major histocompatibility complex. Genomics.

[42] Horton R, Wilming L, Rand V, Lovering RC, Bruford EA, Khodiyar VK, Lush MJ, Povey S, Talbot CC, Wright MW (2004). Gene Map of the Extended Human MHC. Nat Rev Genet.

[43] Jones EY, Fugger L, Strominger JL, Siebold C (2006). MHC class II proteins and disease: a structural perspective. Nat Rev Immunol.

[44] Madden DR (1995). The Three-Dimensional Structure of Peptide-MHC Complexes. Annu Rev Immunol.

[45] Benacerraf B (1981). Role of MHC gene products in immune regulation. Science.

[46] Alfonso C, Karlsson L (2000). Nonclassical MHC Class II Molecules. Annu Rev Immunol.

[47] Kupfermann H, Satta Y, Takahata N, Tichy H, Klein J (1999). Evolution of Mhc-DRB introns: implications for the origin of primates. J Mol Evol.

[48] Mayer F, Brunner A (2007). Non-neutral evolution of the major histocompatibility complex class II gene DRB1 in the sac-winged bat Saccopteryx bilineata. Heredity.

[49] Richman AD, Herrera LG, Ortega-Garcia S, Flores-Martinez JJ, Arroyo-Cabrales J, Morales-Malacara JB (2010). Class II DRB polymorphism and sequence diversity in two vesper bats in the genus *Myotis*. Int J Immunogenet.

[50] Schad J, Dechmann DKN, Voigt CC, Sommer S (2012). Evidence for the ‘Good Genes’ Model: Association of MHC Class II *DRB* Alleles with Ectoparasitism and Reproductive State in the Neotropical Lesser Bulldog Bat, *Noctilio albiventris*. PLoS ONE.

[51] Real-Monroy MD, Martínez-Méndez N, Ortega J (2014). MHC-DRB Exon 2 Diversity of the Jamaican Fruit-Eating Bat (*Artibeus jamaicensis*) from Mexico. Acta Chiropt.

[52] Salmier A, de Thoisy B, Crouau-Roy B, Lacoste V, Lavergne A (2016). Spatial pattern of genetic diversity and selection in the MHC class II DRB of three Neotropical bat species. BMC Evol Biol.

[53] Schad J, Dechmann DKN, Voigt CC, Sommer S (2011). MHC class II DRB diversity, selection pattern and population structure in a neotropical bat species, Noctilio albiventris. Heredity.

[54] Schad J, Voigt C, Greiner S, Dechmann D, Sommer S (2012). Independent evolution of functional MHC class II *DRB* genes in New World bat species. Immunogenetics.

[55] Ng JHJ, Tachedjian M, Deakin J, Wynne JW, Cui J, Haring V, Broz I, Chen H, Belov K, Wang L-F, Baker ML (2016). Evolution and comparative analysis of the bat MHC-I region. Sci Rep.

[56] Zhang G, Cowled C, Shi Z, Huang Z, Bishop-Lilly KA, Fang X, Wynne JW, Xiong Z, Baker ML, Zhao W (2013). Comparative Analysis of Bat Genomes Provides Insight into the Evolution of Flight and Immunity. Science.

[57] Altschul SF, Gish W, Miller W, Myers EW, Lipman DJ (1990). Basic local alignment search tool. J Mol Biol.

[58] Burge C, Karlin S (1997). Prediction of complete gene structures in human genomic DNA. J Mol Biol.

[59] Sullivan MJ, Petty NK, Beatson SA (2011). Easyfig: a genome comparison visualiser. Bioinformatics.

[60] Schneider TD, Stephens RM (1990). Sequence logos: a new way to display consensus sequences. Nucleic Acids Res.

[61] Tamura K, Peterson D, Peterson N, Stecher G, Nei M, Kumar S (2011). MEGA5: Molecular Evolutionary Genetics Analysis using Maximum Likelihood, Evolutionary Distance, and Maximum Parsimony Methods. Mol Biol Evol.

[62] Edgar R (2004). MUSCLE: a multiple sequence alignment method with reduced time and space complexity. BMC Bioinf.

[63] Marsh SGE, Parham P, Barber LD. 11 - HLA POlymorphism, Peptide-binding Motifs and T-Cell Epitopes. In: The HLA FactsBook. London: Academic Press; 2000:61-72.

[64] Bondinas GP, Moustakas AK, Papadopoulos GK (2007). The spectrum of HLA-DQ and HLA-DR alleles, 2006: a listing correlating sequence and structure with function. Immunogenetics.

[65] Felsenstein J (1981). Evolutionary trees from DNA sequences: A maximum likelihood approach. J Mol Evol.

[66] Felsenstein J (1985). Confidence Limits on Phylogenies: An Approach Using the Bootstrap. Evolution.

[67] Waddell PJ, Steel MA (1997). General Time-Reversible Distances with Unequal Rates across Sites: Mixing Γ and Inverse Gaussian Distributions with Invariant Sites. Mol Phylogenet Evol.

[68] Saitou N, Nei M (1987). The neighbor-joining method: a new method for reconstructing phylogenetic trees. Mol Biol Evol.

[69] Rzhetsky A, Nei M (1992). A Simple Method for Estimating and Testing Minimum-Evolution Trees. Mol Biol Evol.

[70] Brodie R, Smith A, Roper R, Tcherepanov V, Upton C (2004). Base-By-Base: Single nucleotide-level analysis of whole viral genome alignments. BMC Bioinf.

[71] Klein J, Bontrop R, Dawkins R, Erlich H, Gyllensten U, Heise E, Jones P, Parham P, Wakeland E, Watkins D (1990). Nomenclature for the major histocompatibility complexes of different species: a proposal. Immunogenetics.

[72] Belov K, Deakin JE, Papenfuss AT, Baker ML, Melman SD, Siddle HV, Gouin N, Goode DL, Sargeant TJ, Robinson MD (2006). Reconstructing an ancestral mammalian immune supercomplex from a marsupial major histocompatibility complex. PLoS Biol.

[73] Gao J, Liu K, Liu H, Blair H, Li G, Chen C, Tan P, Ma R (2010). A complete DNA sequence map of the ovine Major Histocompatibility Complex. BMC Genomics.

[74] Lunney JK, Ho C-S, Wysocki M, Smith DM (2009). Molecular genetics of the swine major histocompatibility complex, the SLA complex. Dev Comp Immunol.

[75] Smith JDL, Gregory TR (2009). The genome sizes of megabats (Chiroptera: Pteropodidae) are remarkably constrained. Biol Lett.

[76] Papenfuss A, Baker M, Feng Z-P, Tachedjian M, Crameri G, Cowled C, Ng J, Janardhana V, Field H, Wang L-F (2012). The immune gene repertoire of an important viral reservoir, the Australian black flying fox. BMC Genomics.

[77] Wan Q-H, Zeng C-J, Ni X-W, Pan H-J, Fang S-G (2009). Giant Panda Genomic Data Provide Insight into the Birth-and-Death Process of Mammalian Major Histocompatibility Complex Class II Genes. PLoS ONE.

[78] Siddle H, Deakin J, Coggill P, Wilming L, Harrow J, Kaufman J, Beck S, Belov K (2011). The tammar wallaby major histocompatibility complex shows evidence of past genomic instability. BMC Genomics.

[79] van Ham SM, Tjin EPM, Lillemeier BF, Grüneberg U, van Meijgaarden KE, Pastoors L, Verwoerd D, Tulp A, Canas B, Rahman D (1997). HLA-DO is a negative modulator of HLA-DM-mediated MHC class II peptide loading. Curr Biol.

[80] Dorn A, Fehling HJ, Koch W, Le Meur M, Gerlinger P, Benoist C, Mathis D (1988). B-cell control region at the 5’ end of a major histocompatibility complex class II gene: sequences and factors. Mol Cell Biol.

[81] Benoist C, Mathis D (1990). Regulation of Major Histocompatibility Complex Class-II Genes: X, Y and Other Letters of the Alphabet. Annu Rev Immunol.

[82] Ting JP-Y, Trowsdale J (2002). Genetic Control of MHC Class II Expression. Cell.

[83] Cheng Y, Stuart A, Morris K, Taylor R, Siddle H, Deakin J, Jones M, Amemiya C, Belov K (2012). Antigen-presenting genes and genomic copy number variations in the Tasmanian devil MHC. BMC Genomics.

[84] Cogswell JP, Basta PV, Ting JP (1990). X-box-binding proteins positively and negatively regulate transcription of the HLA-DRA gene through interaction with discrete upstream W and V elements. Proc Natl Acad Sci.

[85] Reith W, Satola S, Sanchez CH, Amaldi I, Lisowska-Grospierre B, Griscelli C, Hadam MR, Mach B (1988). Congenital immunodeficiency with a regulatory defect in MHC class II gene expression lacks a specific HLA-DR promoter binding protein, RF-X. Cell.

[86] Dorn A, Bollekens J, Staub A, Benoist C, Mathis D (1987). A multiplicity of CCAAT box-binding proteins. Cell.

[87] Dorn A, Durand B, Marfing C, Le Meur M, Benoist C, Mathis D (1987). Conserved major histocompatibility complex class II boxes--X and Y--are transcriptional control elements and specifically bind nuclear proteins. Proc Natl Acad Sci.

[88] Steimle V, Otten LA, Zufferey M, Mach B (1993). Complementation cloning of an MHC class II transactivator mutated in hereditary MHC class II deficiency (or bare lymphocyte syndrome). Cell.

[89] Deakin JE, Siddle HV, Cross JGR, Belov K, Graves JAM (2007). Class I genes have split from the MHC in the tammar wallaby. Cytogenet Genome Res.

