# Supplementary material for: Insights into the ancestral organisation of the mammalian MHC class II region from the genome of the pteropid bat, Pteropus alecto
Source: BMC Genomics. 2017 May 18;18:388. doi: 10.1186/s12864-017-3760-0 (PMC5437515; doi:10.1186/s12864-017-3760-0)
Supplement: Supplementary file 1 — Previously Reported P. alecto MHC-II Transcriptome Sequences and Their Correspondence to Loci. (DOC 33 kb) [file 12864_2017_3760_MOESM1_ESM.doc]

**Additional file 1.** Previously Reported *P. alecto* MHC-II Transcriptome Sequences and Their Correspondence to Loci.

| Bat MHC-II Locus | Bat Transcriptome Transcript |
| --- | --- |
| ***Ptal-DMA*** | Locus25_15339_Transcript_1/2 |
| ***Ptal-DOA*** | Locus31mer_8595_Transcript_1/1 |
| ***Ptal-DQA1*** | Locus31mer_645_Transcript_1/1 |
| ***Ptal-DQA2*** | Locus27_784_Transcript_4/7 |
| ***Ptal-DRA*** | Locus31_2258_Transcript_1/1 |
| ***Ptal-DMB*** | Locus25_854_Transcript_1/1 |
| ***Ptal-DOB*** | Locus23_36201_Transcript_1/1 |
| ***Ptal-DQB1*** | Locus27_1995_Transcript_2/5 |
| ***Ptal-DRB1*** | Locus29_225_Transcript_6/9 |
| **Transcripts from Papenfuss *et al.* 2012** | |
