# Supplementary material for: Insights into the ancestral organisation of the mammalian MHC class II region from the genome of the pteropid bat, Pteropus alecto
Source: BMC Genomics. 2017 May 18;18:388. doi: 10.1186/s12864-017-3760-0 (PMC5437515; doi:10.1186/s12864-017-3760-0)

**Additional file 2.** Amino acid alignment of bat (*Ptal*) MHC-II A genes, (A) *DMA*, (B) *DOA*, (C) *DQA* and (D) *DRA*, against various mammals. Dashes indicate identical residues; Dots indicate gaps; P Peptide-Binding Sites; 4 CD4 Interaction Sites; C Cysteine residues in α2 domain likely to form intra-chain disulphide bonds. Percentage similarity of sequences within a particular α domain is reflected at the end of their respective sequences, with reference to the top sequence in the alignment. Red and blue represent percentage similarity of nucleotides and amino acid residues respectively within the stated α1 and α2 domains. *Hosa – Homo sapiens* ; *Eqca* – *Equus caballus; Susc – Sus scrofa; Mumu* – *Mus musculus*.


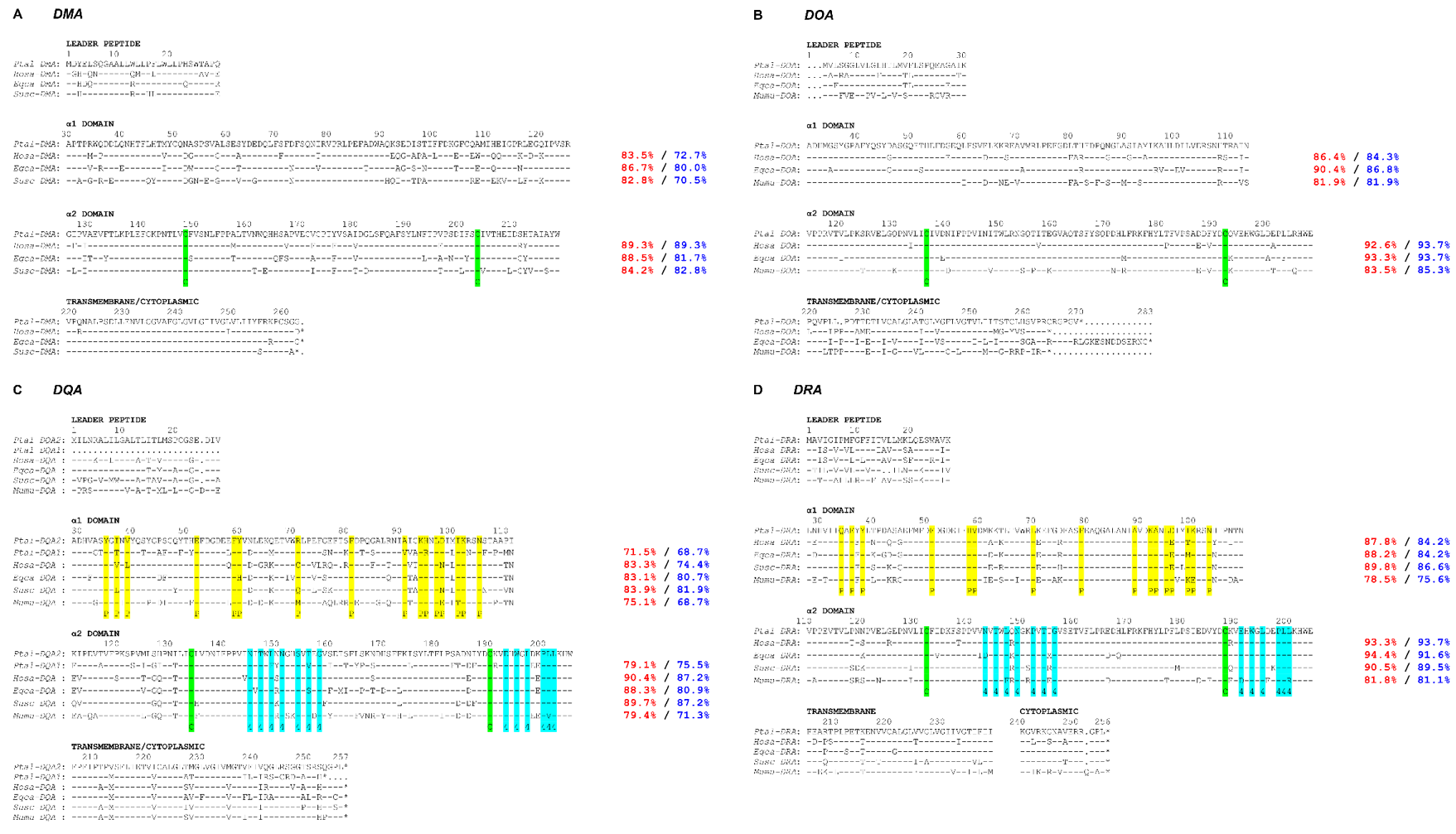

Supplement: Supplementary file 2 — Amino acid alignment of bat (Ptal) MHC-II A genes, (A) DMA, (B) DOA, (C) DQA and (D) DRA, against various mammals. Dashes indicate identical residues; Dots indicate gaps; P Peptide-Binding Sites; 4 CD4 Interaction Sites; C Cysteine residues in α2 domain likely to form intra-chain disulphide bonds. Percentage similarity of sequences within a particular α domain is reflected at the end of their respective sequences, with reference to the top sequence in the alignment. Red and blue represent percentage similarity of nucleotides and amino acid residues respectively within the stated α1 and α2 domains. Hosa – Homo sapiens; Eqca – Equus caballus; Susc – Sus scrofa; Mumu – Mus musculus. (DOC 412 kb) [file 12864_2017_3760_MOESM2_ESM.doc]
