# Supplementary material for: Insights into the ancestral organisation of the mammalian MHC class II region from the genome of the pteropid bat, Pteropus alecto
Source: BMC Genomics. 2017 May 18;18:388. doi: 10.1186/s12864-017-3760-0 (PMC5437515; doi:10.1186/s12864-017-3760-0)

**Additional file 4.** Amino acid alignment of bat (*Ptal*) antigen-processing (AP) genes, (A) *PSMB8*, (B) *PSMB9*, (C) *TAP1*, (D) *TAP2* and (E) *TAPBP*, against human (*Hosa*), mouse (*Mumu*) and horse (*Eqca*). Dashes indicate identical residues; Dots indicate gaps. Percentage similarity of sequences is reflected at the end of their respective sequences, with reference to the top sequence (*Ptal*) in the alignment. Red and blue represent percentage similarity of nucleotides and amino acid residues respectively.


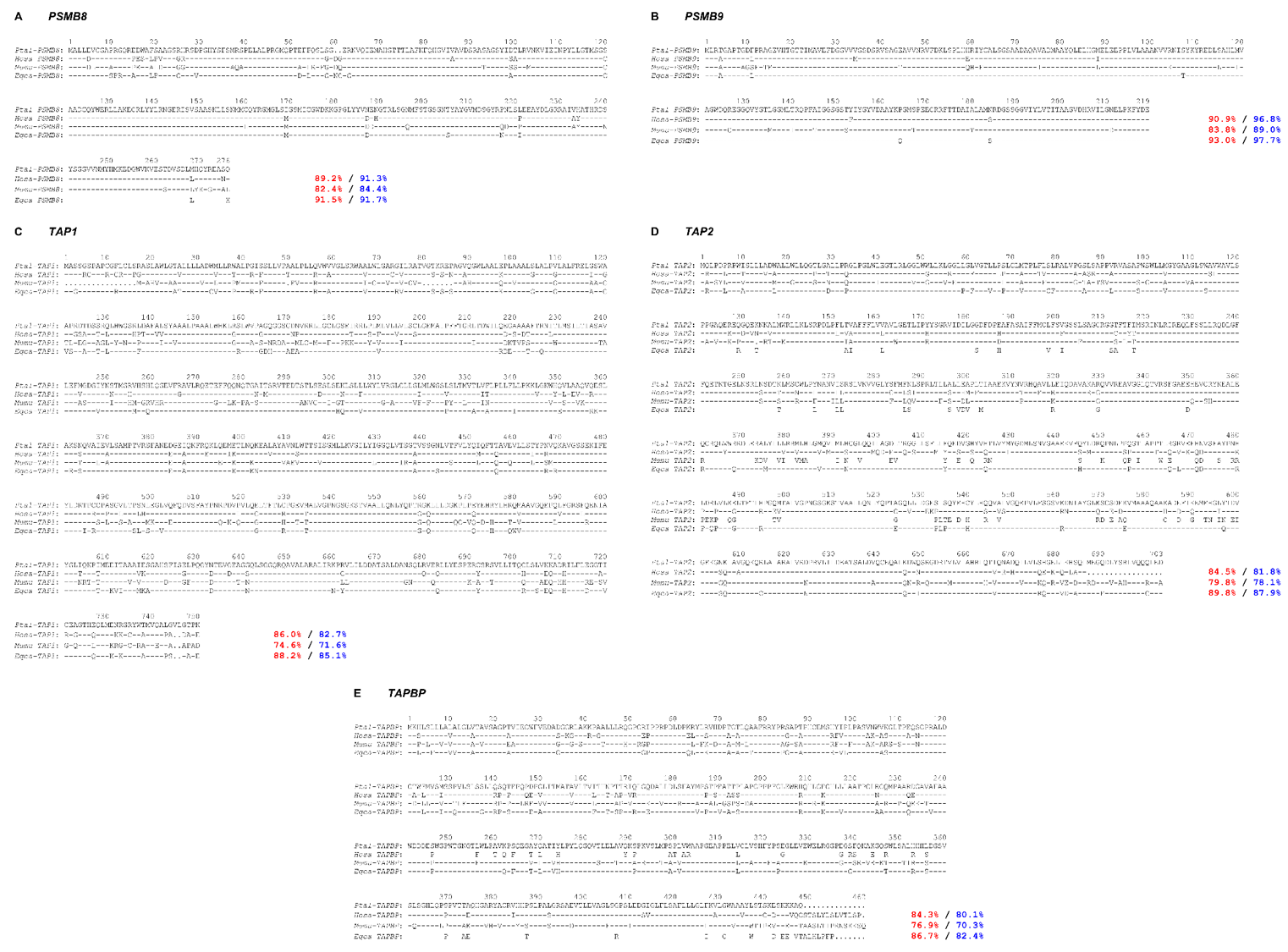

Supplement: Supplementary file 4 — Amino acid alignment of bat (Ptal) antigen-processing (AP) genes, (A) PSMB8, (B) PSMB9, (C) TAP1, (D) TAP2 and (E) TAPBP, against human (Hosa), mouse (Mumu) and horse (Eqca). Dashes indicate identical residues; Dots indicate gaps. Percentage similarity of sequences is reflected at the end of their respective sequences, with reference to the top sequence (Ptal) in the alignment. Red and blue represent percentage similarity of nucleotides and amino acid residues respectively. (DOC 434 kb) [file 12864_2017_3760_MOESM4_ESM.doc]
